# Small Intestinal Bacterial Overgrowth in Children: Clinical Features and Treatment Response

**DOI:** 10.1097/PG9.0000000000000185

**Published:** 2022-03-17

**Authors:** Maria Isabel Peinado Fabregat, Rebecca M. Gardner, Maheen A. Hassan, Kristopher Kapphahn, Ann Ming Yeh

**Affiliations:** From the * Department of Pediatrics, Division of General Pediatrics at Stanford University, Stanford, CA; † Quantitative Sciences Unit, Stanford School of Medicine, Stanford, CA; ‡ Department of Pediatrics, Division of Gastroenterology, Hepatology, and Nutrition at Stanford University, Stanford, CA.

**Keywords:** overgrowth, intestinal overgrowth, breath test, BT, SIBO

## Abstract

To characterize the population of children diagnosed with small intestinal bacterial overgrowth (SIBO) based on breath test (BT), correlate symptomatology, and describe SIBO treatments and treatment efficacy.

**Methods:**

A retrospective cohort study of pediatric patients seen at Stanford Children’s Health Gastroenterology Clinics from 2012 to 2018 who had a positive BT, defined by a rise in hydrogen by ≥20 ppm, a baseline hydrogen level ≥20 ppm, or a methane value ≥10 ppm. The main outcome was symptom resolution, defined as complete or partial improvement after a course of treatment. Absolute standardized differences and Chi-square tests were used to assess associations.

**Results:**

From 98 children, 54 met inclusion and did not meet exclusion criteria (53.7% female). Lactulose substrate was used for 41 (75.9%) patients, whereas glucose was used for 13 (24.1%). Complete or partial resolution of symptoms was achieved in 13 of 16 (81.2%) patients who received probiotics with or without antibiotics versus 21 of 31 (67.7%) patients treated with antibiotics alone (*P* = 0.524). Metronidazole versus rifaximin versus other antibiotics showed no significant difference in symptom resolution (12 (63.2%), 13 (76.5%), 7 (77.8%), respectively, *P* = 0.601).

**Conclusion:**

Seventy-two percent of patients experienced at least partial symptom relief after treatment. We did not find a strong correlation between specific symptoms and analyte elevation. There was no difference in effectiveness between metronidazole and rifaximin to treat SIBO symptoms. Further research needs to be done to determine effective treatments for SIBO in pediatrics.

**What do we know?**

- Children with SIBO present with a variety of symptoms including abdominal pain, bloating, diarrhea, and constipation.
- There are no current guidelines on treatment and evaluation of treatment success.

**What is new?**

- The majority of children (72.3%) with SIBO had partial or complete resolution of symptoms after treatment.
- Symptoms of SIBO often recur and 22.2% of the patients required one or more subsequent courses of antibiotics.

## INTRODUCTION

Small intestinal bacterial overgrowth (SIBO) is defined as excessive numbers of bacteria within the small bowel causing gastrointestinal symptoms (1). The symptomatology in SIBO is broad, ranging from mild bloating to enteropathy, which in turn may cause malabsorption and malnutrition. The most common symptoms include abdominal pain, distention, flatulence, burping, and diarrhea. The incidence is estimated to be 0–35% in healthy children (2).

While the gold standard to diagnose SIBO is a jejunal aspirate culture (3), this is not always practical in pediatrics. The Carbohydrate Breath Test (BT) is the predominant noninvasive test used to diagnose SIBO. Glucose and lactulose BTs, when compared with jejunal aspirate cultures, showed sensitivities of 60–70% and specificities of 40–80% for SIBO diagnosis (4).

Poor standardization regarding methodology and interpretation of results has led to different definitions of a positive BT. According to the North American Consensus from the *American Journal of Gastroenterology* a rise of ≥20 ppm from baseline in hydrogen by 90 minutes or a level of ≥10 ppm of methane is considered positive when using either glucose or lactulose substrate (4).

In the pediatric population, data are lacking regarding the efficacy of antibiotics for SIBO and which antibiotic regimen is more effective. Studies in children have been limited by small groups of specific populations, such as chronic abdominal pain, irritable bowel syndrome (IBS), and malnutrition (5–7). In adults, studies report that rifaximin and neomycin are effective for IBS, especially when combined (8), but insufficient evidence exists for children. As a result, the selection of antibiotics is often secondary to insurance coverage, cost, and medication tolerance (9).

The aims of this study were to retrospectively characterize the population of children diagnosed with SIBO based on BT results, correlate clinical symptomatology with abnormal readings in BT, and describe SIBO treatment efficacy.

## METHODS

This is an IRB-approved retrospective cohort study of pediatric patients diagnosed with SIBO seen at Stanford Children’s Health Gastroenterology Clinics from June 2012 to October 2018.

This research used data or services provided by STARR, “STAnford medicine Research data Repository,” a clinical data warehouse containing live Epic data from Stanford Health Care, the Stanford Children’s Hospital, the University Healthcare Alliance and Packard Children’s Health Alliance clinics and other auxiliary data from hospital applications such as radiology PACS. STARR platform is developed and operated by Stanford Medicine Research IT team and is made possible by Stanford School of Medicine Research Office.

Inclusion criteria were: (1) patients of age 1–21 years who had a positive BT using either lactulose or glucose found through STARR database. Exclusion criteria were: (1) patients with concomitant infectious gastroenteritis at the time of diagnosis and (2) patients that used antibiotics or probiotics in the past month before the BT. A positive BT was defined by a baseline hydrogen level ≥20, a rise in hydrogen by 20 parts per million (ppm) or higher above basal level, or a value ≥10 ppm of methane with either glucose or lactulose substrate.

BT protocol at our center required the patient to have nothing to eat or drink 12 hours before the test, no antibiotics or probiotics 2 weeks before the test and brushing teeth with only water the day of the test. Substrate choice was determined by the ordering provider. No specification on the presence or absence of symptoms was noted in the chart that determined the choice of substrate. The dose of glucose was 1 g/kg of weight (max 75 g), and the dose of lactulose was 1 g/kg of weight (max 10 g). This substrate was mixed in 8–10 oz of water before administration. Breath samples were taken at baseline and subsequently every 20 minutes for 3 hours.

A thorough chart review was performed for a total of 98 patients identified as having a positive BT. Fifty-four patients met the inclusion and did not meet exclusion criteria, while the remaining 44 were excluded due to the utilization of fructose or lactose for the BT substrate.

Data collected from the study cohort included:

- Descriptive cohort data
  ◦ Demographic data
  ◦ Presence of comorbidities
  ◦ Risk factors
  ◦ Symptoms reported
  ◦ History of constipation and constipation treatment (if any)
- BT data
  ◦ Substrate used for BT (glucose or lactulose)
  ◦ Baseline and peak levels of hydrogen and methane
- Treatment data
  ◦ Treatment modality (antibiotics, probiotics with or without antibiotics)
  ◦ Type of antibiotic used (rifaximin, metronidazole, cephalexin, amoxicillin–clavulanic acid, neomycin, ciprofloxacin, trimethoprim–sulfamethoxazole)
  ◦ Dose and duration of treatment (7–10 days, >10 days)
  ◦ Number of courses of antibiotics provided, timing between courses
  ◦ Special diet (vegetarian, vegan, low FODMAPS, specific carbohydrate diet, gluten-free, gluten “light”)
- Resolution of symptoms data
  ◦ Presence of subsequent BTs
  ◦ Reported symptom resolution.

The primary outcome was symptom resolution, defined as a complete or partial improvement after a course of treatment. Patients with unknown symptom resolution were not included in the denominator.

### Statistical Analysis

Using baseline and peak levels of hydrogen and methane, we derived an analyte elevation variable defined as hydrogen positive (elevated baseline or rise above baseline), methane positive, or both. A sensitivity analysis comparing patients in the hydrogen baseline group to those in the hydrogen peak group yielded no clinically significant differences between the 2 groups, leading us to combine these groups for the final analysis. Treatment was compared as antibiotics versus probiotics with or without antibiotics. We also compared antibiotic types—rifaximin, metronidazole, and “other antibiotics” which pooled cephalexin, amoxicillin–clavulanic acid, neomycin, ciprofloxacin, and TMP–SMX due to small sample size.

We calculated overall descriptive statistics and stratified by treatment and analyte elevation. Means and SDs are reported for continuous variables, and frequencies and percentages are reported for categorical variables. We compared groups using absolute standardized differences (ASD), a measure of the difference in means or proportions between 2 or more groups divided by the pooled standard deviation and expressed in units of standard deviations (10). ASD values of 0.2, 0.5, and 0.8 correspond to small, moderate, and large differences between the groups, respectively. Chi-square tests were used to calculate associations between treatment type, analyte, and substrate with symptom resolution. All analyses were conducted with R version 3.6.2 (11).

## RESULTS

Of 54 children who met study inclusion and exclusion criteria, 53.7% were female, 46.3% were male. Table 1 summarizes the demographics (age and sex), comorbidities, risk factors, symptoms for which the test was performed, and other clinical characteristics. Abdominal pain, bloating, and constipation were the most frequent symptoms (72.2%, 53.7%, and 50%, respectively). The most common SIBO risk factors present in our cohort included a history of gastrointestinal infection in the last year and the use of proton pump inhibitors in the last month (18.5% for both risk factors). Only 7.4% (n = 4) had previous intestinal surgeries which included only major procedures such as bowel resection. Endoscopic procedures, feeding tubes, or gastric fundoplication were not counted as major intestinal surgery. Comorbidities such as spinal neurological abnormalities, immunodeficiency syndromes, thyroid disease, inflammatory bowel disease, diabetes mellitus, chronic liver disease, cystic fibrosis, and pancreatitis were evaluated, and zero subjects presented with such conditions.

**Table 1.** Overall cohort

	Overall
n	54
Female, n (%)	29 (53.7)
Age, mean (SD)	11.33 (4.51)
Comorbidities, n (%)	
IBS	6 (11.1)
Celiac disease	5 (9.3)
Short bowel syndrome	4 (7.4)
Transplant	1 (1.9)
Intestinal motility disorder (MMIH, EDS, dysmotility)	2 (3.7)
Risk factors, n (%)	
Altered mucosal barrier	3 (5.6)
Previous use of PPI (1 month)	10 (18.5)
Previous use of antibiotics (1 month)	6 (11.1)
Recurrent antibiotic use (>3 courses in last year)	1 (1.9)
Previous intestinal surgeries	4 (7.4)
Gastrointestinal infection (last year)	10 (18.5)
Travel (last year)	3 (5.6)
Symptoms, n (%)	
Diarrhea	18 (33.3)
Bloating	29 (53.7)
Nausea	16 (29.6)
Dyspepsia	2 (3.7)
Abdominal pain	39 (72.2)
Constipation	27 (50)
Flatulence	17 (31.5)
Burping	6 (11.1)
Constipation Treatment, n (%)	23 (42.6)
Substrate, n (%)	
Lactulose	41 (75.9)
Glucose	13 (24.1)
Special diet, n (%)	28 (51.9)
Analyte elevation, n (%)	
Hydrogen	28 (51.9)
Hydrogen and methane	23 (42.6)
Methane only	3 (5.6)
Treatment, n (%)	
Antibiotics	34 (63.0)
Probiotics	2 (3.7)
Both	17 (31.5)
None	1 (1.9)
Course, n (%)	
7–10 days	30 (58.8)
12–14 days	15 (29.4)
21 days	3 (5.9)
Other	3 (5.9)
Subsequent Breath Test, n (%)	16 (29.6)
Reason for Subsequent Test, n (%)	
Persistence of symptoms	12 (75.0)
Assessment of treatment efficacy	2 (12.5)
New symptoms	2 (12.5)
Subsequent Test Negative, n (%)	3 (18.8)
Symptom Resolution, n (%)	
Not resolved	13 (27.7)
Yes or partially improved	34 (72.3)
Missing values*	7 (13.0)

EDS = Ehlers-Danlos syndromes; IBS = Irritable bowel syndrome; MMIH = megacystis microcolon intestinal hypoperistalsis syndrome.

*Missing values are not included in calculation of percentages for nonmissing categories.

Lactulose was the substrate used for 41 (75.9%) tests, whereas glucose was the substrate for 13 (24.1%). Patients tested using a lactulose substrate had similar symptom resolution rates compared to those who used a glucose substrate (27 (75%) vs 7 (63.6%), *P* = 0.725). Sixteen (29.6%) patients had a subsequent BT, and most of these (75%) were performed due to persistence of symptoms. Table 2 summarizes demographics and other clinical factors stratified by treatment, antibiotic type, and analyte elevation. Table 3 shows symptom resolution stratified by treatment, antibiotic type, and analyte elevation.

**Table 2.** Stratified analyses (treatment, antibiotic type, and analyte elevation)

	Probiotics with/without antibiotics	Antibiotics only	ASD*	Metronidazole	Rifaximin	Other	ASD*	Hydrogen	Methane with/without Hydrogen	ASD*
n	19	34		21	20	10		28	26	
Female, n (%)	8 (42.1)	20 (58.8)	0.339	14 (66.7)	8 (40.0)	5 (50.0)	0.367	18 (64.3)	11 (42.3)	0.452
Age, mean (SD)	12.21 (3.26)	11.03 (5.01)	0.279	11.86 (4.8)	10.35 (4.69)	12.90 (2.92)	0.411	12.18 (4.85)	10.42 (4.01)	0.395
Symptoms, n (%)										
Diarrhea	6 (31.6)	12 (35.3)	0.079	9 (42.9)	5 (25.0)	3 (30.0)	0.255	11 (39.3)	7 (26.9)	0.265
Bloating	9 (47.4)	19 (55.9)	0.171	9 (42.9)	11 (55.0)	8 (80.0)	0.541	14 (50.0)	15 (57.7)	0.155
Nausea	4 (21.1)	12 (35.3)	0.321	5 (23.8)	6 (30.0)	5 (50.0)	0.374	10 (35.7)	6 (23.1)	0.28
Dyspepsia	0 (0.0)	2 (5.9)	0.354	1 (4.8)	1 (5.0)	0 (0.0)	0.217	0 (0.0)	2 (7.7)	0.408
Abdominal pain	15 (78.9)	23 (67.6)	0.258	15 (71.4)	13 (65.0)	8 (80.0)	0.227	19 (67.9)	20 (76.9)	0.204
Constipation	9 (47.4))	17 (50.0)	0.053	13 (61.9)	9 (45.0)	3 (30.0)	0.444	14 (50.0)	13 (50.0)	<0.001
Flatulence	10 (52.6)	6 (17.6)	0.788	6 (28.6)	4 (20.0)	6 (60.0)	0.587	8 (28.6)	9 (34.6)	0.13
Burping	3 (15.8)	3 (8.8)	0.213	2 (9.5)	2 (10.0)	2 (20.0)	0.199	4 (14.3)	2 (7.7)	0.212
Constipation treatment, n (%)	8 (42.1)	14 (41.2)	0.019	12 (57.1)	6 (30.0)	3 (30.0)	0.379	13 (46.4)	10 (38.5)	0.162
Glucose substrate, n (%)	4 (21.1)	9 (26.5)	0.128	3 (14.3)	6 (30.0)	4 (40.0)	0.4	11 (39.3)	2 (7.7)	0.803
Special diet, n (%)	13 (68.4)	15 (44.1)	0.505	9 (42.9)	9 (45.0)	8 (80.0)	0.548	16 (57.1)	12 (46.2)	0.221
Treatment, n (%)			1.819				0.354			0.329
Antibiotics	0 (0.0)	34 (100.0)		14 (66.7)	15 (75.0)	5 (50.0)		17 (60.7)	17 (65.4)	
Probiotics	2 (10.5)	0 (0.0)		0 (0.0)	0 (0.0)	0 (0.0)		1 (3.6)	1 (3.8)	
Both	17 (89.5)	0 (0.0)		7 (33.3)	5 (25.0)	5 (50.0)		10 (35.7)	7 (26.9)	
None	0 (0.0)	0 (0.0)		0 (0.0)	0 (0.0)	0 (0.0)		0 (0.0)	1 (3.8)	
Course, n (%)			0.443				0.846			0.212
7–10 days	8 (47.1)	22 (64.7)		11 (52.4)	16 (80.0)	3 (30.0)		15 (55.6)	15 (62.5)	
12–14 days	6 (35.3)	9 (26.5)		8 (38.1)	3 (15.0)	4 (40.0)		8 (29.6)	7 (29.2)	
21 days	2 (11.8)	1 (2.9)		1 (4.8)	0 (0.0)	2 (20.0)		2 (7.4)	1 (4.2)	
Other	1 (5.9)	2 (5.9)		1 (4.8)	1 (5.0)	1 (10.0)		2 (7.4)	1 (4.2)	
Subsequent Breath Test, n (%)	4 (21.1)	12 (35.3)	0.321	5 (23.8)	9 (45.0)	2 (20.0)	0.368	6 (21.4)	10 (38.5)	0.378
Reason for Subsequent Test, n (%)			0.756				1.097			0.305
Persistence of symptoms	3 (75.0)	9 (75.0)		3 (60.0)	7 (77.8)	2 (100)		4 (66.7)	8 (80.0)	
Assessment of treatment efficacy	0 (0.0)	2 (16.7)		2 (40.0)	0 (0.0)	0 (0.0)		1 (16.7)	1 (10.0)	
New symptoms	1 (25.0)	1 (8.3)		0 (0.0)	2 (22.2)	0 (0.0)		1 (16.7)	1 (10.0)	
Subsequent Test Negative, n (%)	1 (25.0)	2 (16.7)	0.206	0 (0.0)	1 (11.1)	2 (100.0)	NaN	1 (16.7)	2 (20.0)	0.086

ASD = absolute standardized differences.

*Absolute Standardized Difference; values of 0.2, 0.5, and 0.8 correspond to small, medium, and large differences.

**Table 3.** Symptom resolution stratified by treatment, antibiotic type, and analyte elevation

	Probiotics with/without antibiotics	Antibiotics only	*P* value	Metronidazole	Rifaximin	Other	*P* value	Hydrogen	Methane with/without Hydrogen	*P* value
n*	19	34		21	20	10		28	26	
Symptom resolution, n (%)			0.524				0.601			0.209
Not resolved	3 (18.8)	10 (32.3)		7 (36.8)	4 (23.5)	2 (22.2)		8 (32.0)	5 (22.7)	
Yes or partially improved	13 (81.2)	21 (67.7)		12 (63.2)	13 (76.5)	7 (77.8)		17 (68.0)	17 (77.3)	
Unknown**	n = 3	n = 3		n = 2	n = 3	n = 1		n = 3	n = 4	

*Analysis included n = 54. Note that 1 patient did not receive any treatment (probiotics and/or antibiotics) and 3 patients did not receive antibiotics.

**Unknown values are not included in calculation of *P* values nor percentages for nonmissing categories.

Because short bowel syndrome or intestinal motility disorder is diagnosed associated with SIBO, we performed a sensitivity analysis excluding these 6 patients for our primary outcome of symptom resolution. Symptom resolution stratified by treatment, antibiotic type, and analyte elevation in this group was similar to that of the whole cohort (See Supplemental Digital Content Table 1, http://links.lww.com/PG9/A74).

### Analyte Elevation

Twenty-eight patients demonstrated an elevation of hydrogen only, whereas 26 patients demonstrated methane positivity with or without elevated hydrogen (Supplemental Digital Content Table 2 for available values, http://links.lww.com/PG9/A75).

Diarrhea and nausea were more prevalent in the hydrogen only group versus the methane positive group (39.3% vs 26.9%, ASD = 0.265, and 35.7% vs 23.1% ASD = 0.28, respectively). Constipation was equally prevalent in both groups (50%, ASD = <0.001).

Of the 26 patients with methane elevation, only 2 patients (7.7%) were in the glucose substrate group, while the rest of the 24 patients were in the lactulose substrate group (92.3%).

Patients who had methane positive tests (with or without hydrogen elevation) were more likely to receive a subsequent BT (10 (38.5%) vs 6 (21.4%), ASD = 0.378). However, symptom resolution was similar between the 2 groups of hydrogen vs. methane positivity (17 (68%) vs 17 (77.3%), *P* = 0.209).

### Treatment

Regarding treatment, 34 (63%) patients used antibiotics only, 2 (3.7%) used probiotics only, 17 (31.5%) used both antibiotics and probiotics, and 1 patient (1.9%) received no treatment. Of those treated with antibiotics (94.4% of our cohort), 20 (39.2%) received rifaximin, 21 (41.2%) received metronidazole, and 10 (19.6%) were prescribed another antibiotic. Rifaximin dosing ranged from 100 mg twice a day to 550 mg three times a day. Metronidazole dosing ranged from 180 mg three times a day to 500 mg three times a day. Antibiotic treatment length ranged from 7 to 14 days. Of those with documented symptom improvement after treatment, 13 (81.2%) patients treated with probiotics (with or without antibiotics) had complete or partial resolution of symptoms versus 21 (67.7%) patients treated only with antibiotics (*P* = 0.524). Use of metronidazole versus rifaximin versus other antibiotics also showed no statistically significant difference in efficacy for resolving SIBO symptoms (12 (63.2%), 13 (76.5%), 7 (77.8%), *P* = 0.601). Symptom resolution was not different whether patients were treated with metronidazole, rifaximin or other antibiotics when stratified by hydrogen elevation (8 (61.5%), 3 (75%), 5 (71.4%), *P* = 0.839) versus methane with or without hydrogen elevation (4 (66.7%), 10 (76.9%), 2 (100%), *P* = 0.628).

Of the 54 patients who had positive BTs, 10 (18.5%) received 1 repeat course of antibiotics, 1 (1.9%) received 3 courses of antibiotics, and 1 patient (1.9%) received 4 courses of antibiotics during the study period. Thirty-four (72.3%) patients had complete or partial symptom resolution after treament (7 patients were lost to follow-up). We found no difference in symptom resolution amongst patients treated with antibiotics for 7–10 days vs. >10 days (15 (71.4%) vs. 17 (70.8%), *P* = 1.00).

In total, 16 patients received a repeat BT: 12 (75%) for persistence of symptoms after treatment, 2 (12.5%) for assessment of treatment efficacy, and 2 (12.5%) for new symptoms. Children treated with rifaximin had higher retesting rates for persistence of symptoms. Seven of 9 (77.8%) children in the rifaximin group, who received a subsequent BT, were retested for reasons of persistence of symptoms compared to 3 of 5 (60%) children retested in the metronidazole group. Of the 16 with repeat BTs, 3 were negative. Two of these patients with negative repeat tests had recurrent symptoms; 1 had new symptoms of epigastric pain.

## DISCUSSION

SIBO is an emerging condition in pediatric gastroenterology that is garnering more attention in otherwise healthy patients. Lack of data regarding its prevalence in the general pediatric population along with imperfect diagnosis methodology makes it a challenging condition to understand and treat adequately. Currently, BTs encompass the main noninvasive method to diagnose SIBO.

### Breath Test and Analyte Elevation

In our study, patients that received a lactulose BT were more likely to have elevated methane than those who received a glucose BT. This may be secondary to lactulose reaching the colon early, causing a false positive methane elevation secondary to colonic fermentation (12). BTs rely on the assumption that the substrate will remain in the small bowel for the duration of the study cutoff—90 minutes. The wide range of bowel transit time in children, with many having a small bowel transit time under 90 minutes, limits the specificity of lactulose as a substrate (13).

Further, our study found patients with methane positivity were more likely to have a follow-up BT than those with hydrogen elevation only (10 (38.5%) versus 6 (21.4%)), usually due to persistence of symptoms (8 (80%)). This finding might suggest that SIBO with methane positivity is associated with a more refractory course or higher recurrence rate.

### Clinical Presentation

Unlike previous studies that have documented a correlation between constipation and methane elevation (14–16), we did not find a strong correlation between specific symptoms and analyte elevation. However, there was a small correlation between the presence of diarrhea and nausea with elevation of hydrogen.

As mentioned previously, patients with methane positivity were more likely to have a follow-up BT than those with hydrogen elevation only usually due to persistence of symptoms. One hypothesis is that methane production is secondary to constipation and possibly delayed colonic transit, and that constipation needs to be treated to correct the dysbiosis. While no reports show that constipation and dysmotility management decreases methanogen burden, studies in adults show methane production is more prevalent in those with constipation (17). In pediatrics, methane production has been shown to positively correlate with prolonged whole intestinal transit time (14).

Studies do not show a correlation of symptom severity and hydrogen or methane peak values, but we have 2 interesting cases to report. One patient had very high levels of hydrogen (peak level 148 ppm) with predominantly abdominal pain who had resolution of symptoms with treatment purely based on probiotics *(Lactobacillus rhamnosis GG*). Another patient, with very high levels of hydrogen (peak level 138 ppm), presented with significant bloating and constipation and had resolution of symptoms after a course of rifaximin. However, he had a recurrence of symptoms and received a repeat BT which was again positive for SIBO. His repeat BT showed an improved peak level of hydrogen (55 ppm), but higher baseline level of hydrogen and methane positivity from his prior test. This suggests that the first course of antibiotics only partially treated the SIBO, especially given the significantly elevated peak. However, further research would need to investigate the immediate efficacy of the antibiotic course with a BT within 1 month of antibiotic completion.

The prevalence of SIBO in IBS is an ongoing area of study. Patients with IBS have dysbiosis which is thought to increase intestinal permeability, dysmotility, chronic inflammation, and possibly alter enteral neuronal activity (18). Seven patients in our cohort had a diagnosis of IBS at the time of BT. IBS diagnosis was based on clinical evaluation by the provider. One patient was diagnosed as IBS-diarrhea predominant (IBS-D) and the rest were diagnosed as IBS-constipation predominant (IBS-C). The patient with IBS-D had a positive BT with a baseline and peak hydrogen of 2 ppm and 59 ppm, respectively. All 6 patients with IBS-C had complaints of gas, bloating, or excessive burping. BT results were heterogeneous and did not show a clear pattern of positivity (Table 4): 3 had an elevated hydrogen peak, 1 had an isolated hydrogen baseline, 1 had an elevated methane level, and 1 had an elevated hydrogen baseline, peak, and methane level. Further, 3 of these patients had markedly elevated hydrogen peaks of >50. These results suggest that patients with significant IBS-C with bloating should undergo SIBO screening tests. These findings echo prior studies where patients with functional gastrointestinal disorders concomitantly tested positive for SIBO on glucose BT (14.3–17.2%) or lactulose BT (53.4%) (5,19). We were unable to assess if these patients would have still met the criteria for IBS after SIBO was treated. This was unable to be fully assessed in our study due to the retrospective study design, as some of these patients did not want to treat the SIBO, and others were lost to follow-up and/or had incomplete documentation.

**Table 4.** Pattern of analyte elevation for each IBS patient

	Hydrogen elevated baseline	Hydrogen elevated peak	Elevated methane ≥10
IBS # 1		x	
IBS # 2	x		
IBS # 3		x	
IBS # 4		x	
IBS # 5			x
IBS # 6	x	x	x
IBS # 7	x		x

IBS = Irritable bowel syndrome.

### Treatment and Symptom Resolution

Our study suggests no difference in effectiveness between metronidazole and rifaximin to treat SIBO symptoms. Despite antibiotic choice with or without probiotics, 72% of patients experienced some degree of symptom relief. To date, no published prospective trials compare antibiotic effectiveness on SIBO in the pediatric population, and prior studies show mixed results. A double-blind, placebo-controlled trial found no changes in abdominal pain in children with SIBO after being treated with rifaximin (20), while another study found rifaximin effective for treating SIBO in children with IBS (21). A study in 20 SIBO patients treated with trimethoprim-sulfamethoxazole and metronidazole for 14 days demonstrated symptom relief was associated with decreased hydrogen production, not methane, suggesting that antibiotic therapy may differ in effectiveness based on analyte elevation (22). This was a small study where other infectious gastrointestinal diseases may have also played a role in treatment efficacy. It is important to note that these prior studies often lack a control group, were directed to specific subpopulations and had small sample sizes (23). Our study also had too small of a sample size to make conclusions of antibiotic efficacy based on analyte elevation. Therefore, a prospective head-to-head trial looking at antibiotic effectiveness for specific subtypes of SIBO may help elucidate more targeted SIBO treatment.

Literature regarding the treatment of symptom recurrence is very scarce. Pimentel et al studied 51 adults with systemic sclerosis treated with cyclic courses of antibiotics for SIBO to prevent recurrence and found that repeated treatment for 3 consecutive months yielded 52% eradication of SIBO (assessed by glucose breath testing) and improvement of symptoms (1). In our study, 16 patients received a repeat BT for symptom recurrence and only 3 of these were negative. Clinically, if symptoms recur, it would be reasonable to repeat BT to determine if there is SIBO recurrence, visceral hypersensitivity, or other new etiology.

Our study had several limitations, including the retrospective design and small sample size. Substrate choice for BT and SIBO treatment was largely provider-dependent. We were unable to perform a robust analysis of glucose and lactulose data separately due to small sample size. Future studies should evaluate substrates separately given that their metabolism in the intestine differs. Current test preparation guidelines were published after this cohort was started (1,4). Insufficient preparation could have led to a number of patients who had elevated hydrogen levels at baseline.

Other limitations include a wide variance in the timeline of presentation, diagnosis, treatment, and follow-up. Although many patients were on a special diet, the retrospective nature of the study did not allow for analysis to determine whether diet had a causative effect on SIBO symptoms. Patients did not automatically repeat a BT after a course of treatment to determine treatment efficacy in normalizing the BT. Interpretation of resolution of symptoms (as a proxy for effective SIBO treatment) was limited to chart review and subsequent follow-up. As seen in practice, patients that present with a resolution of symptoms find no further need to follow-up, biasing the results towards treatment failure.

In conclusion, our retrospective single-center study indicates that children with SIBO present with a variety of symptoms including abdominal pain, bloating, diarrhea and constipation. Although initial antibiotic treatment is successful in the majority of children (63–78%, depending on the antibiotic used), 22.2% of our cohort still required one or more repeat courses of antibiotics, indicating a high SIBO treatment failure or recurrence rate. The myriad of limitations including its diverse presentation and the patient’s comorbidities makes SIBO a challenging diagnosis to be studied with precision.

Further prospective research, with head-to-head comparisons of antibiotic regimens, and perhaps utilizing newer technology to adequately sample microbiota and gas production in the small bowel, may be needed to determine more sensitive and specific diagnostic testing for SIBO as well as determine more targeted treatments for SIBO in pediatrics.

## ACKNOWLEDGMENTS

Quantitative Sciences Unit, Department of Medicine, Stanford School of Medicine for assistance in statistical review. Stanford Children’s Health, Division of Pediatric Gastroenterology, Registered Nurses for assistance in breath test protocols. Dr. Peinado Fabregat is an Ernest and Amelia Gallo Endowed Postdoctoral Fellow. The work described in this article was supported by the Stanford Maternal and Child Health Research Institute.
